# Mini Review: New Treatments in Psoriatic Arthritis. Focus on the IL-23/17 Axis

**DOI:** 10.3389/fphar.2019.00872

**Published:** 2019-08-06

**Authors:** Lazaros I. Sakkas, Efterpi Zafiriou, Dimitrios P. Bogdanos

**Affiliations:** ^1^Department of Rheumatology and Clinical Immunology, Faculty of Medicine, School of Health Sciences, University of Thessaly, Larissa, Greece; ^2^Department of Dermatology, Faculty of Medicine, School of Health Sciences, University of Thessaly, Larissa, Greece

**Keywords:** anti-IL-17, cytokine, IL-17, monoclonal antibodies, psoriatic disease

## Abstract

Psoriasis, an inflammatory skin disease, and psoriatic arthritis (PsA), an inflammatory arthritis, share clinical, genetic, and pathogenic factors and may be summed as one disease, the psoriatic disease. Interleukin (IL)-17 plays a major role in the development of both psoriasis and PsA. IL-23 is important in the proliferation and maintenance of IL-17, and therefore, cytokines of the IL-23/IL-17 axis attracted much interest as therapeutic targets in psoriasis and PsA. Therapeutic agents targeting the IL-23/IL-17 axis have been proven to be very effective in psoriasis and PsA, some are already in the therapeutic armamentarium and others are in the development. Some agents, target IL-23 and others IL-17 and include anti-IL-12/IL-23 p40 (ustekinumab, briankizumab), anti-IL-23p19 (guselkumab, tildrakizumab, risankizumab, brazikumab, mirikizumab), anti-IL-17A (secukinumab, ixekizumab), dual anti-IL-17A and anti-IL-17F (bimekizumab), or anti-IL-17 receptor (brodalumab) monoclonal antibodies. Janus tyrosine kinase(JAK) inhibitors also directly affect IL-23 and, thus, IL-17. After the first-generation pan-JAK inhibitors have been shown efficacy (tofacitinib, baricitinib), new-generation selective JAK inhibitors (filgotinib, upadacitinib) are under investigation in psoriasis and PsA.

## Introduction

Psoriatic arthritis (PsA) is an inflammatory joint disease associated with psoriasis, a common inflammatory skin disease with a prevalence 2% to 3% worldwide (Rachakonda et al., 2014). PsA occurs in a third of the patients with psoriasis (Mease et al., 2013) and apart from psoriasis, manifests with peripheral arthritis, enthesitis, dactylitis, spine involvement, and uveitis. The presence of enthesitis, which may be subclinical is very common in psoriasis and substantially increase the frequency of PsA in patients with psoriasis (Sakkas et al., 2019; Mease et al., 2019b) The pathogeneses of both PsA and psoriasis are incompletely understood, but innate and adaptive cells and proinflammatory cytokines are involved, particularly the interleukin(IL)-23/IL-17 axis (Sakkas and Bogdanos, 2017). The shared pathophysiological and clinical features of psoriasis and PsA have led some investigators to consider these two entities as one, the psoriatic disease. Recent studies, reporting autoreactive T cells recognizing LL37, ADAMTSL5, and PLA2G4D (phospholipase A2 group IVD) and producing interleukin(IL)-17 (Lande et al., 2014; Cheung et al., 2016; Hawkes et al., 2017) bring forward the autoimmune element in the pathogenesis of psoriatic disease.

### IL-17 Cytokine

The nature of IL-17 has been described in detail elsewhere (Wright et al., 2008 and **Supplementary Text** in gray) The IL-17 family consists of six proteins that share homology among them, and are known as IL-17A, IL-17B, IL-17C, IL-17D, IL-17E, and IL-17F. Among IL-17 family members, IL-17F shares the strongest amino acid sequence with IL-17A whereas IL-17E (IL-25) is the most distant from IL-17A. The IL-17 receptor differs from other cytokine receptors and consist of five members, IL-17RA, IL-17RB, IL-17RC, IL-17RD, and IL-17RE (Chang and Dong, 2011; Veldhoen, 2017). IL-17A and IL-17F are secreted by the same cell types, as homodimers or IL-17A/IL-17F heterodimers and signal through the constitutively expressed IL-17RA paired with the inducible IL-17RC (Wright et al., 2008).

IL-17 is produced by T (Th17) cells, γδ T cells, natural killer T (NKT) cells, NK cells, and type 3 innate lymphoid cells, which also can produce IL-17F, and IL-22. Th17 cells are differentiated from naïve T cells by the action of any of these three cytokine combinations, IL-6 and TGFβ, IL-21 and TGFβ, or IL-6, IL-23, and IL-1β (Veldhoen, 2017). The expression of IL-22 can be regulated separately. IL-22 is induced by IL-23 and signals through the IL-22Rα/IL-10Rβ heterodimer. IL-23 consists of p40 (which is also a subunit of IL-12, IL-12p40) and p19 subunit (IL-23p19) and signals through its receptor IL-23R paired with IL-12Rβ1 and is required for the proliferation and survival of Th17 cells (Teng et al., 2015).

IL-17 *in vitro* can induce the production of proinflammatory cytokines, such as IL-6, IL-1, GM-CSF, G-CSF, and enhances the expression of several chemokines involved in chemoattraction of neutrophils, monocytes, and lymphocytes. IL-17A and IL-17F induce similar cytokine profiles with IL-17F being less effective in macrophage cytokine production and act in synergy with TNFα. However, they may also have distinct roles. In a colitis model caused by dextran sulfate sodium, IL-17A deficiency enhanced colitis, whereas IL-17F deficiency reduced colitis. In addition, in an asthma model, IL-17A deficiency reduced Th2 responses, whereas IL-17F deficiency enhanced Th2 responses (Yang et al., 2008).

IL-25(IL-17E) is produced by eosinophils, mast cells, basophils, epithelial cells, and signals through IL-17RA paired with IL-17RB to promote Th2 cell immune responses. IL-17C signals through the IL-17RA/IL-17RE complex in Th17 cells and promotes proinflammatory responses (Chang et al., 2011). IL-17D is preferentially expressed in skeletal muscles, adipose tissue, and brain and induces IL-8 and IL-6 production in endothelial cells but inhibits hemopoiesis (Starnes et al., 2002). IL-17B and IL-17C are proinflammatory cytokines, as they exacerbate collagen-induced arthritis in mice (Yamaguchi et al., 2007).

### IL-23/IL-17 Axis in Psoriatic Disease

IL-17A and IL-17F as well as their receptor complex (IL17RA/IL-17RC) are expressed in psoriatic skin lesion (Johansen et al., 2009) and PsA synovial tissues (van Baarsen et al., 2014; Glatt et al., 2018).

IL-17-producing T (Th17) cells are major players in the pathogenesis of psoriasis because they are present in psoriatic lesions and can induce activation/proliferation of keratinocytes and endothelial cells (Lowes et al., 2013; Skepner et al., 2014; Sakkas and Bogdanos, 2017; Boutet et al., 2018). Furthermore, T cells recognizing skin autoantigens, such as LL-37, ADAMTSL5, and PLA2G4D, produce IL-17 (Lande et al., 2014; Cheung et al., 2016; Hawkes et al., 2017). IL-17 synergizes with TNFα to induce the production of proinflammatory cytokines IL-6, IL-8, IL-1β (Wang et al., 2013). A mice model with T cell-specific hyperactive STAT3 has augmented Th17 response and exhibits epidermal proliferation and synovial-entheseal inflammation which improve by abrogation of IL-17 and IL-22 cytokines (Yang et al., 2018). Similarly, mannan from *Saccharomyces cerevisiae* (Baker’s yeast) administered by intraperitoneal injection to mice induces a PsA-like disease with joint inflammation and psoriasis-like skin lesions, which are prevented by neutralization of IL-17A (Khmaladze et al., 2014). IL-17- and IL-22-producing cells are present with different frequencies in various anatomical sites with IL-22 expression being very low in arthritic joints in PsA (Benham et al., 2013). IL-23 is produced locally at entheseal sites, and IL-23-induced IL-22 is found to be critical for the development of enthesitis (Sherlock et al., 2012).

### Treatments Targeting the IL-23/IL-17 Axis

**Figure 1:**
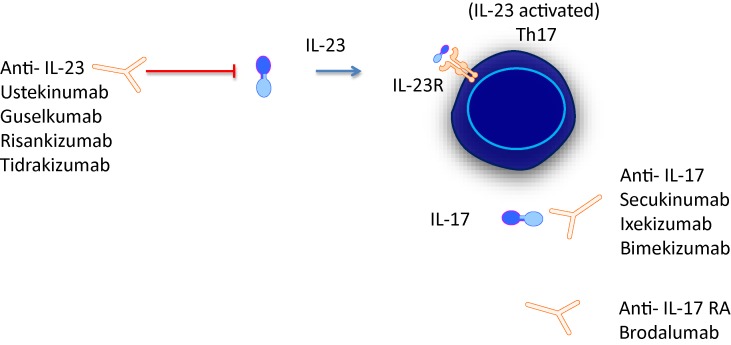
IL-23/IL-17 axis is the focus of novel treatments targeting either cytokine or cytokine receptors.

The first biologicals that target the IL-23/IL-17 axis approved for psoriatic disease were ustekizumab and secukinumab. List of all agents and an illustrated agents are provided in **Figure 1** and **Table 1**.

**Table 1:** Major clinical trials of trreatments targeting the IL-23/IL-17 axis and their main efficacy and serious adverse events characteristics in patients with psoriatic arthritis.

Agent	Biologic target	Trial	ACR50 Week12/16	ACR50 Week24	SAEs (%)	Reference
Ustekinumab	Anti-IL-12/23 p40 MoAb	PSUMMIT 1 Phase III		90 mg 27.9% 45 mg 24.9% Placebo 8.7%	1.5% 2% 2%	McInnes et al., 2013
Ustekinumab	Anti-IL-12/23 p40 MoAb	PSUMMIT 2 Phase III		90 mg 22.9% 45 mg 17.5% Placebo 6.7%	1.9% 0% 4.8%	Ritchlin et al., 2014
Guselkunab	Anti-IL-23 p19 MoAb	Phase II		100 mg 34% Placebo 10%	6% 0%	Deodhar et al., 2018
Secukinumab	Anti-IL-17A MoAb	FUTURE-1 Phase III		150 mg 34.7% 75 mg 30.7% Placebo 7.4%	11.5% 7.4% 5%	Mease et al., 2015
Secukinumab	Anti-IL-17A MoAb	FUTURE-2 Phase III		300 mg 35% 150 mg 35% Placebo 7% Anti-TNFIR 300 mg 27% Anti-TNFIR 150 mg 19% Placebo 9% Anti-TNFnaive 300 mg 39% Anti-TNFnaive 150 mg 44% Placebo 6%	6.4% 5.1% 8.6%	McInnes et al., 2015
Secukinumab	Anti-IL-17A MoAb	FUTURE-5 Phase III	300mg with loading dose 39.6% 150mg with loading dose 35.9% 150mg without loading dose 32% Placebo 8.1%		3.2% 4.1% 2.7% 3.6%	Mease et al., 2018b
Ixekizumab	Anti-IL-17A MoAb	SPIRIT-P1 Phase III	q2w:39.8% qw4:33.6% Placebo:4.7% Adalimuab:29.7%	q2w:46.6% q4w:40.2% Adalimumab:38.6%	q2w:4.9% q4w:3.7% Placebo:1.9%	Mease et al., 2017b
Ixekizumab	Anti-IL-17A MoAb	SPIRIT-P2 Phase III		q2w:33% q4w:35% placebo:5%	Q2w:7% Q4w:3% Placebo:2%	Nash et al., 2017
Brodalumab	Snti-IL17RA MoAb	Phase II	140mg:14% 280mg:14% Placebo:4%		140mg:2% 280mg:4% Placebo:2%	Mease et al., 2014

IL-17RA, interleukin 17 receptor A; MoAb, monoclonal antibody; q2w, once every 2 weeks; q4w, once every 4 weeks; SAEs, serious advese events.

#### A. IL-12/IL-23p40 Inhibitors

**Ustekinumab**. Ustekinumab is a human IgG1 MoAb that binds with high affinity to the p40 subunit of IL-12 and IL-23 and is approved for the treatment of moderate to severe psoriasis and PsA. Ustekinumab has a mean half-life of 20 days but its effect stay much longer than expected from its half-life (Gottlieb et al., 2007). The dose is 45 mg (90 mg in persons >100 kg) given subcutaneously (SC) at weeks 0, 4, and then every 12 weeks. Ustekinumab showed efficacy in psoriasis in two phase III trials (PHOENIX-1 and PHOENIX-2) (Leonardi et al., 2008; Papp et al., 2008) and in two phase III trials in PsA (PSUMMIT-1 and PSUMMIT-2) (McInnes et al., 2013; Ritchlin et al., 2014). At week 24, ustekinumab achieved American College of Rheumatology (ACR) 50 in 26.4% of patients versus 8.7% of patients in the placebo group. In TNFα experienced patients ustekinumab achieved ACR20 in 35.6% of patients versus 14.5% in the placebo group. By week 52, PASI 75 and PASI 100 was achieved by 60.6% and 43.7% of patients, respectively. Dactylitis, enthesitis, and spondylitis were also improved, and radiographic joint damage was inhibited (Kavanaugh et al., 2014). Ustekinumab has a good safety profile, with nasopharyngitis being the most common adverse effect, whereas major cardiovascular events and carcinoma were rare events (Ghosh et al., 2019).

**Briakinumab**. Briakinumab is a fully human IgG1 MoAb that binds to the p40 subunit of IL-12 and IL-23 and has a terminal half-life of 9 days. In a dose ranging phase II trial in psoriasis, briakinumab at week 12 achieved PASI75 in ∼90% of patients versus 3% in the placebo group, but caused adverse effects in 36% of patients versus 10% in the placebo group (Kimball et al., 2008).

#### B. IL-23p19 Inhibitors

**Tildrakizumab**. Tildrakizumab is a humanized IgG1κ MoAb that binds to the p19 subunit of IL-23 with high affinity and inhibits downstream signaling of IL-23. After SC injection, its half-life is 25 days (Khalilieh et al., 2018). It has been approved for the treatment of moderate-to severe plaque psoriasis. In two phase III trials (reSURFACE 1 and reSURFACE 2), patients with psoriasis were randomized to receive Tildrakizumab 200 or 100 mg SC at weeks 0, 4, and 16, or placebo (reSURFACE-1), and tildrakizumab 200 mg, or 100 mg at weeks 0,1, and 16, or placebo or etanercept (reSURFACE-2). At 12 weeks in the reSURFACE-1 trial, 62% of the tildrakizumab 200 mg group, 64% of the tildrakizumab 100 mg group, and 6% of the placebo achieved PAS75. In the reSURFACE-2 trial, the respective figures were 66%, 61%, 6%, and 48% of the etanercept group. Tildrakizumab was well-tolerated with a low frequency of adverse effects (Reich et al., 2017). At 28 weeks, 78% of patients achieved PASI75, 58% achieved PASI90, and 29% achieved PASI100 (Sinclair and Thirthar Palanivelu, 2018).

**Guselkumab**. Early reports in mice with collagen-induced arthritis mice showing that a loss of IL-23 gene(p19^−/−^) was protective whereas a loss of IL-12 gene(p35^−/−^) exacerbated arthritis (Murphy et al., 2003) lead to efforts to neutralize only IL-23 without affecting IL-12. Guselkumab is a human IgG1λ MoAb that binds to IL-23p19 subunit and inhibits the downstream signaling of IL-23. Its mean half-life is 12 to 19 days (Zhuang et al., 2016). Guselkumab proved to be efficacious treatment for psoriasis in phase III trials (VOYAGE-1 and VOYAGE-12). Patients with psoriasis were randomized to receive guselkumab 100 mg SC at weeks 0, 4, and then every 8 weeks, or placebo or adalimumab 80 mg at week 0, 40 mg at week 1, and then 40 mg every 2 weeks. At week 16, 73.3% of patients achieved PASI90 and 37.4% of patients achieved PASI100 in the guselkumab group compared with 2.9% and 0.6%, respectively, in the placebo group. Adalimumab achieved PASI90 in 49.7% and PASI100 in 17.1% of patients (Blauvelt et al., 2017b). Guselkumab was superior to adalimumab through week 48 (Blauvelt et al., 2017b). Nonresponders to adalimumab at week 28 switched to guselkumab, and 66.1% of them achieved PASI90 at week 48 (Reich K, JAAD 2017;76:418). Guselkumab has a good safety profile and has been approved for the treatment of moderate to severe psoriasis at a dose of 100 mg SC at weeks 0, 4, and then every 8 weeks.

In PsA, guselkumab also appears to be very effective. In a phase II trial, patients with PsA received guselkumab 100 mg SC at weeks 0, 4, and then every 8 weeks. At week 24, guselkumab achieved ACR50/70 in 34%/14% of patients versus 10%/2% in the placebo group and PASI75/90 in 79%/66% of patients versus 13%/6% in the placebo group. Neutropenia was found in 5% of patients as in other anti-IL-17 agents, secukinumab, ixekizumab, and brodalumab (Deodhar et al., 2018).

**Risankizumab**. Risankizumab is a humanized IgG1κ MoAb that targets the IL-23p19 subunit and selectively inhibits IL-23. Its half-life is 27 days (Suleiman et al., 2019). Risankizumab has been tried in psoriasis with impressive efficacy, and a trial in PsA is underway. In a dose ranging trial in psoriasis risankizumab (90 or 180 mg) or ustekinumab were given SC at weeks 0, 4, and 16. At week 12, risankizumab (90 and 180 mg grouped together) achieved PASI90 in 77% of patients compared with 40% of patients in the ustekinumab group (Papp et al., 2017). In two-phase III trials, patients with psoriasis were randomized to receive risankizumab 150 mg, placebo, or ustekinumab. At week 16, risankizumab achieved PASI90 in 75% of patients, placebo in 2% to 4.9% and ustekizumab in 42% to 47% of patients (Gordon et al., 2018). Nasopharyngitis was the most common adverse effect, whereas basal cell carcinoma and acute myocardial infarction have been reported (Papp et al., 2017).

#### C. IL-17 Inhibitors

**Secukinumab**. Secukinumab is a fully human IgG1κ MoAb that selectively binds to IL-17A with high affinity and reduces inflammation. It also quickly restores serum Dkk-1 (Wnt signaling antagonist) in PsA (Fassio et al., 2019). A large body of evidence from many studies including phase III trials FUTURE-1 (Mease et al., 2015) and FUTURE-2 (McInnes et al., 2015) have shown that secukinumab is very effective treatment in patients with PsA, both TNFα naïve and TNFα experienced. It is approved for the treatment of psoriasis, PsA, and ankylosing spondylitis. Dose for PsA: 150 mg SC at week 0, 1, 2, 3, 4, and then every 4 weeks. In PsA with coexistent moderate to severe psoriasis, the dose is 300 mg given as above. If a patient continues to have active arthritis, a dose of 300 mg may be considered.

In a randomized study, secukinumab proved superior to ustekinumab in clearing psoriasis. At week 16, secukinumab achieved PASI90 in 79% and PASI100 in 44.3% of patients compared with 57.6% and 28.4% of patients, respectively, of the ustekinumab group (Thaci et al., 2015). Analysis of results from the FUTURE-2 trial revealed that at week 16, PASDAS remission plus low disease activity in TNFα naive patients was achieved with secukinumab 300 and 150 mg in 46.2% and 42.9% of patients, respectively, versus 17.5% of patients in the placebo group. In TNFα-experienced patients, the corresponding figures were 22.6% and 19.4% versus 13.3% in the placebo group. Furthermore, remission/low disease activity (LDA) was sustained through 2 years in the secukinumab group (Coates et al., 2018). Secukinumab in TNFα naive PsA patients and clazakizumab (an anti-IL-6 MoAb) appear to be the most effective treatments among biologicals in treating dactylitis (Sondag et al., 2019). A recent FUTURE 5 study of secukinumab in PsA to compare the 300 mg with 150 mg dose on clinical and radiographic response revealed that the 300 mg dose with loading dose achieved the numerically highest efficacy in all end points, particularly in psoriasis improvement. A 150-mg dose without loading dose provided comparable results, although resolution of dactylitis and enthesitis was not significant at 16 weeks (Mease et al., 2018b).

There was no difference in the ACR20/50 response between secukinumab and infliximab during the first 16 weeks of treatment in PsA, but later secukinumab achieved higher responses than infliximab (Strand et al., 2019). Also, secukinumab achieved higher ACR responses than adalimumab through 1 year (Nash et al., 2018b).

In one systematic review and meta-analysis, secukinumab was superior to ustekinumab in TNFα naïve but not in TNFα-experienced PsA patients (Kawalec et al., 2018). Secukinumab greatly improved nail psoriasis, as assessed by NAPSI (Reich et al., 2018). Secukinumab was a cost-effective biological in the treatment of PsA with the highest net monetary benefit than other biologicals in Finland (Purmonen et al., 2018).

**Ixekizumab**. Ixekizumab is a recombinant IgG4κ MoAb antibody that binds, with high affinity to and neutralizes IL-17A. Its half-life is 13 days. In two trials (UNCOVER-1 and UNCOVER-2) patients with chronic plaque psoriasis were randomized to receive ixekizumab 80 mg SC every 2 weeks(q2w) or every 4 weeks(q4w) after a starting dose of 160 mg, or placebo or etanercept. At 12 weeks, PASI 75 was achieved by 89.7% of patients in the ixekizumab q2w group, 77.5% of patients in the ixekizumab q4w group, 2.4% of patients in the placebo group, and 41.6% of patients in the etanercept group. Ixekizumab was well tolerated, and upper respiratory infections and injection site reactions were the most frequently reported adverse effects (Griffiths et al., 2015). Ixekizumab was also well tolerated and exhibited sustained efficacy through week 108 (Blauvelt et al., 2017a).

The efficacy of ixekizumab in PsA was assessed in two phase III trials (SPIRIT-P1 and SPIRIT-P2). In SPIRIT-P1, PsA patients with inadequate response to csDMARDs were randomized to receive ixekizumab 80 mg SC every 2 weeks (q2w), ixekizumab 80 mg q4w, placebo, or adalimumab 40 mg q2w. Both ixekizumab regimens received 160 mg loading dose (Mease et al., 2017b). At week 12, both ixekizumab regimens achieved complete clearing of psoriasis (PASI100) in more patients (q2w:40.7%, q4w: 31.5% of patients) than adalimumab (14.7% of patients), whereas the effect on joints, as assessed by ACR50 was comparable between ixekizumab (q2w, 39.8%; q4w, 33.6% of patients) and adalimumab (29.7% of patients). The effect on nail psoriasis, as assessed by NAPSI, was also comparable between ixekizumab (q2w, 27%; q4w, 20% of patients) and adalimumab (19.7% of patients) (Mease et al., 2017b). In biological-naive PsA patients from the SPIRIT-P1 trial, ixekizumab q2w or q4w achieved comparable ACR50 and ACR70 responses and delayed joint structural damage at 24 weeks irrespective of concomitant csDMARD or MTX use. However, structural joint damage progression was less in patients treated with concomitant csDMARD or MTX (Coates et al., 2017). Similarly, the incidence of moderate/severe AEs was similar to placebo regardless of concomitant csDMARD or MTX use (Coates et al., 2017). In the SPIRIT-P2 trial, ixekizumab was found to be effective in TNFα inhibitor inadequate response PsA patients. At 24 weeks ixekizumab both the q2w and the q4w regimens achieved ARC50 response in 35% of patients compared with 5% of patients in the placebo group (Nash et al., 2017). Similarly, ixekizumab achieved high response rates in psoriasis score (PASI 90:44% in q4w and 50% in q2w regimens, and 12% in placebo) (Nash et al., 2017). In PsA patients with inadequate response or intolerant to TNFα inhibitor, at 24 weeks ixekizumab achieved comparable responses regardless of concomitant csDMARD use (Nash et al., 2018a). Data from SPIRIT-P1 and SPIRT-P2 trials showed that ixekizumab significantly improved dactylitis (ixekizumab q2w, 78%; ixekizumab q4w, 65%; placebo, 24% of patients). The effect on enthesitis was less impressive (Gladman et al., 2019). Injection site reactions and mucocutaneous Candida infection were frequent adverse effects (Mease et al., 2019a).

In treating enthesitis and dactylitis, secukinumab, ustekinumab, or ixekizumab may have similar efficacy as TNFα inhibitors (Mourad and Gniadecki, 2019). However, in treating arthritis secukinumab has the highest efficacy in PsA compared with ixekizumab, and ustekinumab, whereas ustekinumab has the lowest probability for severe adverse effects (Wu et al., 2018).

**Bimekizumab**. Bimekizumab is an IgG1κ humanized MoAb that selectively and potently binds to and neutralizes both IL-17A and IL-17F. Bimekizumab inhibition of both IL-17A and IL-17F suppressed proinflammatory cytokine production and neutrophil chemotaxis *in vitro* more effectively than blockade of either IL-17A or IL-17F alone (Glatt et al., 2018). Its mean half-life is 20 days (Glatt et al., 2017). In a phase IIb dose-ranging trial in patients with psoriasis, bimekizumab was given at doses 64 to 480 mg SC every 4 weeks. At 12 weeks, bimekizumab 160 mg (with 320 mg loading dose) achieved impressive improvement of psoriasis with PASI90 in 75% and PASI100 in 60% of patients (Papp et al., 2018). Adverse effects had no apparent relationship to dose. Mucocutaneous fungal infections were reported in 4.3% of bimekizumab-treated patients and transient grade 2 (nonserious) neutropenia in 2.4% of patients (Papp et al., 2018).

In a proof of concept trial in 39 patients with PsA, multiple doses of bimekizumab at weeks 0, 3, and 6 resulted in a rapid and profound joint and skin responses at week 8 that sustained or improved through week 20. In particular, ACR50 response was 40% at week 8 and 56.7% at week 20 compared with 8.3% and 18.2%, respectively, in the placebo group. Similarly, PASI100 was achieved by 86.7% of patients at week 8 and 73.3% at week 20, compared with 0% in the placebo group (Glatt et al., 2018). Two fungal infections were reported in the bimekizumab group, one oropharyngitis and one vulvovaginitis after bimekizumab infusion (Glatt et al., 2018).

**Brodalumab**. Brodalumab is a human IgG2 anti-IL-17 receptor A (IL-17RA) MoAb that inhibits IL-17A, IL-17F, and IL-17E (IL-25). In a phase II dose-ranging trial in psoriasis, at 12 weeks brodalumab at a dose of 140 mg and 210 mg at weeks 0, 1, 2, and then every 2 weeks achieved PASI75 in 77% and 82% of patients, respectively, compared with 0% in the placebo group. The respective percentages for PASI100 were 38% and 62% (Papp et al., 2012). In two phase III trials (AMAGINE-2 and AMAGINE-3) brodalumab at a dose 210 mg was more effective than ustekinumab at week 12: PASI100 in 37% to 44% versus 19% to 22% of patients (Lebwohl et al., 2015).

In a phase II trial in PsA, brodalumab 140 or 280 mg given SC at week 1, 2, and then every 2 weeks, at 12 weeks achieved ACR50 response in 14% of patients compared with 4% in the placebo group and ACR70 response in 5% of patients (Mease et al., 2014). In the open extension study ACR50 response increased to 33% (Mease et al., 2014). Brodalumab appears to be ineffective for dactylitis (Sondag et al., 2019).

Brodalumab has been approved for the treatment of moderate to severe psoriasis at a dose of 210 mg SC at weeks 0, 1, 2, and then every 2 weeks. Brodalumab increases the risk of infections, and, therefore, vaccinations according to local guidelines are recommended before initiation of brodalumab. It may decrease neutrophil count and can cause suicidal ideation.

### JAK Inhibitors

Janus tyrosine kinase (JAK) inhibitors are small molecules, taken orally, that target JAK and block intracellular cytokine pathways. There are four members of the JAK family JAK1, JAK2, JAK3, and TYK2 that form heterodimers and transmit signals from cytokine receptors of the cell membrane. Tofacitinib, a JAK1/JAK3 inhibitor has shown efficacy in PsA (Gladman et al., 2017; Mease et al., 2017a). Baricitinib, a JAK1/JAK2 inhibitor, in a dose-ranging phase IIb trial in psoriasis at week 12 at a dose 8 mg or 10 mg achieved PASI75 in 43% and 54% of patients, respectively, compared with 17% in the placebo group (Papp et al., 2016).

In a phase II trial (EQUATOR) in PsA filgotinib, a selective JAK1 inhibitor at a dose of 200 mg orally once a day at week 16 achieved ACR50 in 55%, LDA(DAPSA ≤ 14) in 49%, and PASI75 in 45% of patients. The respective percentages in the placebo group were 12%, 15%, and 15%. (Mease et al., 2018a). Other (baricitinib (JAK1/JAK2) and selective (upadacitinib [JAK1] JAK inhibitors are being evaluated in PsA.

## Conclusion

IL-23/IL-17 axis cytokines are important players in the pathogenesis of psoriasis and PsA. Inhibition of IL-23 and IL-17 with MoAbs is a very effective therapy for both psoriasis and PsA. The numbers of these agents are increasing.

## Author Contributions

LS and DB scripted the original manuscript. EZ scripted significant parts of the manuscript. DB designed the artwork. All authors approved the final version of the manuscript.

## Conflict of Interest Statement

The authors declare that the research was conducted in the absence of any commercial or financial relationships that could be construed as a potential conflict of interest.
