# Supplementary material for: Mini Review: New Treatments in Psoriatic Arthritis. Focus on the IL-23/17 Axis
Source: Front Pharmacol. 2019 Aug 6;10:872. doi: 10.3389/fphar.2019.00872 (PMC6691125; doi:10.3389/fphar.2019.00872)
Supplement: Supplementary file 1 [file DataSheet_1.docx]

**Supplementary text**

The nature of IL-17 has been described in detail elsewhere (([Wright et al., 2008](#_ENREF_71) and supplementary text in grey) The IL-17 family consists of 6 proteins that share homology among them, and are known as IL-17A, IL-17B, IL-17C, IL-17D, IL-17E, and IL-17F. Among IL-17 family members, IL-17F shares the strongest amino acid sequence with IL-17A whereas IL-17E (IL-25) is the most distant from IL-17A. The IL-17 receptor, differs from other cytokine receptors and consist of 5 members, IL-17RA, IL-17RB, IL-17RC, IL-17RD, and IL-17RE ([Chang and Dong, 2011](#_ENREF_5); [Veldhoen, 2017](#_ENREF_69)). IL-17A and IL-17F are secreted by the same cell types, as homodimers or IL-17A/IL-17F heterodimers and signal through the constitutively expressed IL-17RA paired with the inducible IL-17RC ([Wright et al., 2008](#_ENREF_71)).

IL-17 is produced by T (Th17) cells, γδ T cells, natural killer T (NKT) cells, NK cells, and type 3 innate lymphoid cells, which also can produce IL-17F, and IL-22. Th17 cells are differentiated from naïve T cells by the action of any of these three cytokine combinations, IL-6 and TGFβ, IL-21 and TGFβ, or IL-6, ~~plus~~ IL-23, and ~~plus~~ IL-1β ([Veldhoen, 2017](#_ENREF_69)). The expression of IL-22 can be regulated separately. IL-22 is induced by IL-23 and signals through the IL-22Rα/IL-10Rβ heterodimer. IL-23 consists of p40 (which is also a subunit of IL-12, IL-12p40) and p19 subunit (IL-23p19) and signals through its receptor IL-23R paired with IL-12Rβ1 and is required for the proliferation and survival of Th17 cells ([Teng et al., 2015](#_ENREF_66)).

IL-17 in vitro can induce the production of proinflammatory cytokines, such as IL-6, IL-1, GM-CSF, G-CSF and enhances the expression of several chemokines involved in chemoattraction of neutrophils, monocytes and lymphocytes. IL-17A and IL-17F induce similar cytokine profiles with IL-17F being less effective in macrophage cytokine production, and act in synergy with TNFα. However, they may also have distinct roles. In a colitis model caused by dextran sulfate sodium, IL-17A deficiency enhanced colitis, whereas IL-17F deficiency reduced colitis. In addition, in an asthma model, IL-17A deficiency reduced Th2 responses, whereas IL-17F deficiency enhanced Th2 responses ([Yang et al., 2008](#_ENREF_75)).

IL-25(IL-17E) is produced by eosinophils, mast cells, basophils, epithelial cells and signals through IL-17RA paired with IL-17RB to promote Th2 cell immune responses. IL-17C signals through the IL-17RA/IL-17RE complex in Th17 cells and promotes proinflammatory responses ([Chang et al., 2011](#_ENREF_6)). IL-17D is preferentially expressed in skeletal muscles, adipose tissue, and brain and induces IL-8 and IL-6 production in endothelial cells but inhibits haemopoiesis ([Starnes et al., 2002](#_ENREF_63)). IL-17B and IL-17C are proinflammatory cytokines, as they exacerbate collagen-induced arthritis in mice ([Yamaguchi et al., 2007](#_ENREF_73)).
